# Wide Nematogenic Azomethine/Ester Liquid Crystals Based on New Biphenyl Derivatives: Mesomorphic and Computational Studies

**DOI:** 10.3390/molecules27134150

**Published:** 2022-06-28

**Authors:** Fowzia S. Alamro, Dina A. Tolan, Ahmed M. El-Nahas, Hoda A. Ahmed, Mohamed A. El-Atawy, Nada S. Al-Kadhi, Saadullah G. Aziz, Mohamed F. Shibl

**Affiliations:** 1 Department of Chemistry, College of Science, Princess Nourah bint Abdulrahman University, P.O. Box 84428, Riyadh 11671, Saudi Arabia; fsalamro@pnu.edu.sa (F.S.A.); nsalkadhi@pnu.edu.sa (N.S.A.-K.); 2 Department of Chemistry, Faculty of Science, Menoufia University, Shebin El-Kom 32512, Egypt; d_tolan2005@yahoo.com (D.A.T.); amelnahas@hotmail.com (A.M.E.-N.); 3 Department of Chemistry, College of Science and Humanities, Prince Sattam bin Abdulaziz University, Alkharj 11942, Saudi Arabia; 4 Department of Chemistry, Faculty of Science, Cairo University, Giza 12613, Egypt; 5 Chemistry Department, College of Sciences, Taibah University, Yanbu 30799, Saudi Arabia; mohamed.elatawi@alexu.edu.eg; 6 Chemistry Department, Faculty of Science, Alexandria University, P.O. Box 426, Ibrahemia, Alexandria 21321, Egypt; 7 Chemistry Department, Faculty of Science, King Abdulaziz University, Jeddah 21589, Saudi Arabia; saziz@kau.edu.sa; 8 Center for Sustainable Development, College of Arts and Sciences, Qatar University, Doha 2713, Qatar

**Keywords:** biphenyl liquid crystals, thermal stability, nematic phase, DFT, optimized structures

## Abstract

The thermal stability and mesomorphic behavior of a new biphenyl azomethine liquid crystal homologues series, (E)-4-(([1,1′-biphenyl]-4-ylmethylene)amino)phenyl 4-(alkoxy)benzoate, In, were investigated. The chemical structures of the synthesized compounds were characterized using FT-IR, NMR, and elemental analyses. Differential scanning calorimetry (DSC) and polarized optical microscopy were employed to evaluate the mesomorphic characteristics of the designed homologues. The examined homologues possessed high thermal stability and broad nematogenic temperature ranges. Furthermore, the homologues were covered by enantiotropic nematic phases. The experimental measurements of the mesomorphic behavior were substantiated by computational studies using the density functional theory (DFT) approach. The reactivity parameters, dipole moments, and polarizability of the studied molecules are discussed. The theoretical calculations demonstrated that as the chain length increased, the polarizability of the studied series increased; while it did not significantly affect the HOMO–LUMO energy gap and other reactivity descriptors, the biphenyl moiety had an essential impact on the stability of the possible geometries and their thermal as well as physical parameters.

## 1. Introduction

To understand the relationship between liquid crystals’ (LCs) chemical structures and mesomorphic properties, there has been extensive research on various homologous series of azomethine/esters [1,2,3,4,5]. Organic compounds’ mesophase stability and temperature range are predominantly results of their molecular shape, i.e., slight changes in the molecular geometry lead to major shifts in the mesomorphic behavior. [6]. In the rigid core of the molecule, the Schiff base (azomethine group) functions as a linking group. In spite of the azomethine group’s stepped structure, its linearity is retained, which makes it more stable and allows the development of the mesophase. [7].

The symmetrical and non-symmetrical configurations of the LC molecule are determined by the number of aromatic rings, the length of the terminal alkyl chains, the nature of the mesogenic core, and the variations in polar spacers in the expanded wings. [8]. It has been reported that various materials with two or three rings are possible, as well as molecules that can have non-symmetrical shapes and core units with one or two distinct connections. [9,10]. Furthermore, the azomethine/ester homologues have recently instructed the identification of twist-bend smectic phases [11,12]. The mesophase behavior of non-symmetrical biphenyl derivatives [13,14] has been recently explored. Moreover, the addition of a new connecting group and a change to the rigid component stabilized the mesomorphic behavior and may have resulted in the development of new phases [15,16].

A twist-bend nematic phase has been observed in several Schiff bases, low-molecular-mass liquid crystals [17,18,19,20]. Recent studies have been conducted on syntheses and research activities in this area [8,21,22,23,24,25]. On the other hand, a wide range of terminal substituents, such as small polar compact substituents and alkoxy chains, are employed [26,27,28,29,30]. Variable mesomorphic properties can be promoted by polar substituents with strong dipole moments [31]. Both mesophase stability and melting temperatures are improved by an increased dipole moment [32]. LC mesophases are influenced by the length of the terminal alkoxy chain in terms of their formation, type, thermal stability, and temperature range. The mesogenic part, terminal, and flexible groups are all chosen when developing new thermotropic LCs [33]. Calamitic LC, therefore, exhibits mainly mesomorphic properties depending on their molecular structure. The physical or thermal properties of the forming mesomorphic material are also influenced by the incorporation of a high polar small size lateral group into the main architecture of the molecule, such as the polarizability and dipole moment [34,35]. As the -CH_2_- and -CH_3_ numbers in the terminal chain change from odd to even in LC mesophases, the conformation of the alkyl terminal chain causes significant changes in the thermal and physical properties; this effect is known as the odd–even effect in a series [36]. In the terminal alkyl chain, compounds with an odd number of C atoms are more flexible than those with an even number of CH_2_ units. As a result of this tendency, LC materials have a less uniform orientation [37]. Furthermore, odd and even numbers of C atoms in the total chain length result in different macroscopic characteristics [37,38]. As a result, the odd–even effect [39] provides a novel option for optimizing mesophase and optical properties.

The current work focused on the biphenyl liquid crystalline derivatives with the azomethine/ester as core linkages to fully understand the structure–property relationship. In more recent structure–property relationship investigations of calamitic LCs, we increased the rigidity part of the molecular structure by increasing the number of benzene rings of rode-like molecules by inserting a biphenyl group into the molecular skeleton. The free rotation in biphenyl rings enhanced the flexibility of the molecules, which inspired us to design a new liquid crystalline series containing the biphenyl moiety to study the effect of the molecular flexibility on the physical and thermal properties of formed mesophases. From this aspect, the main objective of our current research is to synthesize a new geometrical structure consisting of four rigid rings with Schiff base/ester linkages and terminal alkoxy chains of various lengths (n). Three ring analogues were described in our prior study [40], and their mesophase behavior was examined. The new biphenyl series (with four benzene rings) was shown to have wider N temperature ranges than the previously studied three-ring homologues [40]. An experimental and theoretical study was conducted to determine whether the length of the terminal groups and the mesogenic core structure affected the behavior of the mesophase transition of biphenyl homologous, namely (E)-4-(([1,1′-biphenyl]-4-ylmethylene)amino)phenyl 4-(alkoxy)benzoate (**I*n***). 
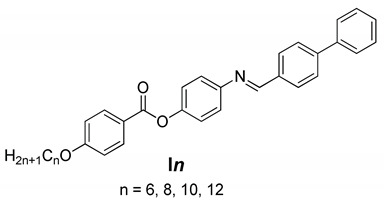


## 2. Results and Discussion

### 2.1. Liquid Crystalline Behavior of Series, **In**

**Figure 1:**
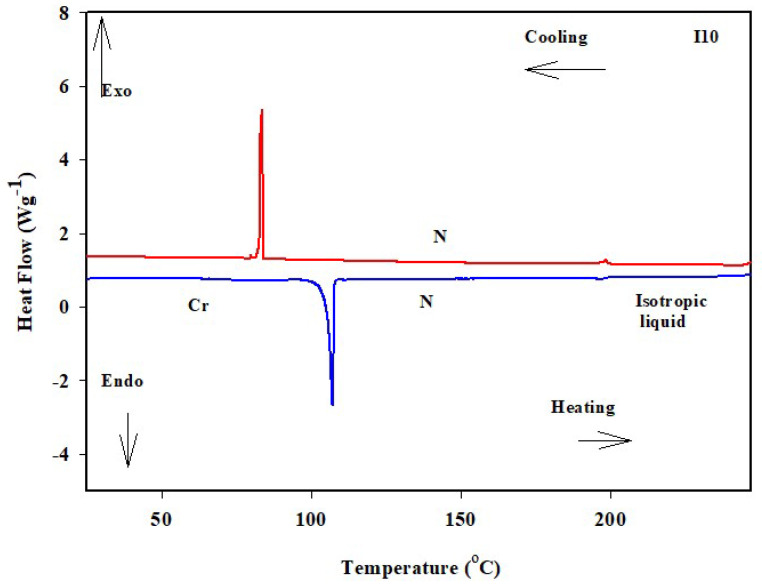
The DSC curve of sample **I10** on heating/cooling rounds at a heating rate of 10 °C min^−1^.

**Figure 2:**
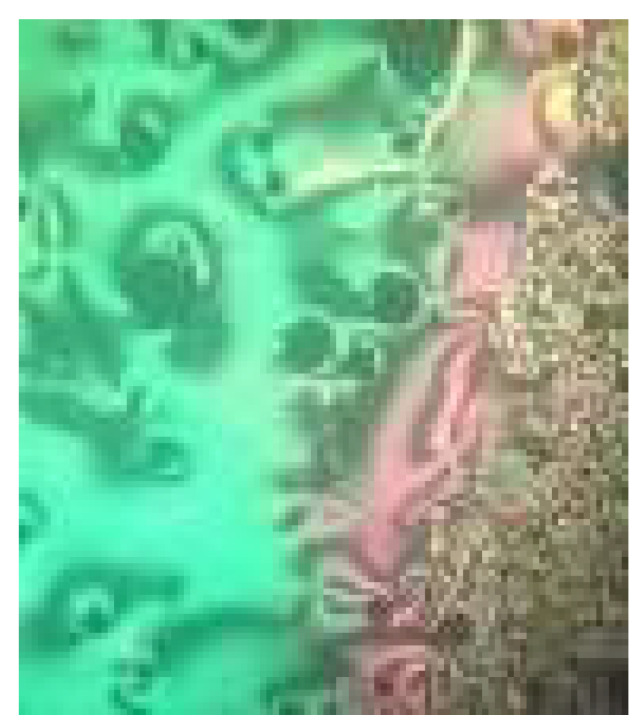
The POM of the N phase texture at 120.0 °C on heating of sample **I10**.

**Figure 3:**
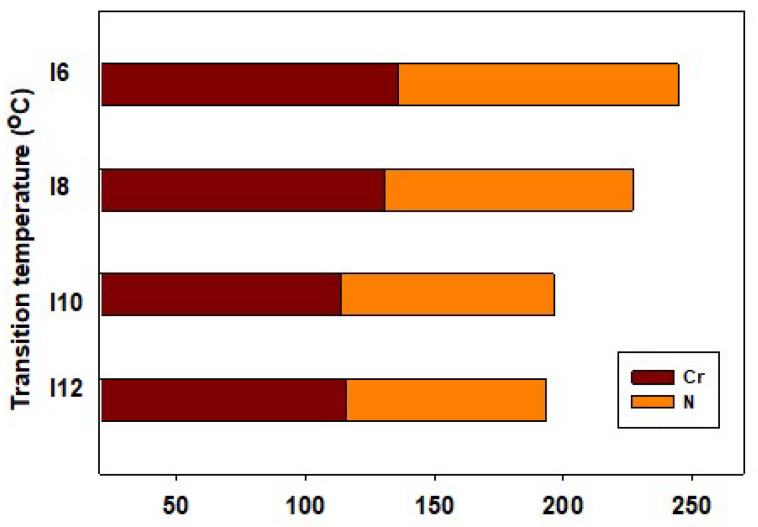
Graphical DSC transitions of the investigated derivatives, **I*n***.

In this study, the mesomorphism of the synthesized series (**I*n***) was examined. According to the measurements made by the DSC, Table 1 collects the transition temperatures and enthalpies obtained. Using the DSC values derived from a second heating/cooling cycle, we tested the stability of the prepared compounds. Figure 1 shows the DSC curve for the synthesized homologue I10, generated by heating and cooling scans. Two endothermic peaks were observed on heating the homologue, which were designated to the Cr-to-N and N-to-isotropic transitions, while two exothermic peaks were seen on cooling, as shown in Figure 1. The DSC data were confirmed by the POM investigations. The DSC data were also corroborated by the POM textures. The POM revealed schlieren textures of the nematic (N) phase (Figure 2). As shown in Figure 1 and Figure 2, all homologues had enantiotropic monomorphic properties. Figure 3 depicts the DSC transition temperatures graphically to examine the terminal flexible chain effect on the mesophase behavior of the investigated structures (**I*n***). As demonstrated in Table 1 and Figure 3, the current series (**I*n***) were all enantiotropic having high thermal transitions and broad mesomorphic ranges. The type of linking groups determines the mesomorphic behavior and the terminal chain length, in general, determines the mesomorphic behavior for any designed LC architecture [41,42,43,44,45]. The melting points (Cr–to–nematic) followed a random pattern, as illustrated in Table 1 and Figure 3. The lowest melting point (Cr-N = 113.5 °C) was observed for homologue **I10**, while the homologue **I6** had the highest melting point (Cr-N = 135.8 °C). The synthesized group exhibited purely N mesophases, and the N phase stability increased with n. The interaction between the mesogenic units was reduced as the chain length increased, lowering the N-I transition temperature, as previously described [43,44,45].

In general, the mesophase behavior is governed to a large extent by the polarity and/or polarizability of the mesogenic core of molecules. The examined mesomorphic temperature range of the **I*n*** series (**ΔT**_N_) decreased with n, as shown in Table 1 and Figure 3. In comparison to the other members, homologue **I6** had the highest nematogenic temperature range and stability, whereas homologue **I12** had the shortest N temperature range and thermal stability. The present results revealed that the stability of the produced mesophases increased with increasing the molecular anisotropy, which resulted from changing the mesogenic core of the molecule. On the other hand, the nature, stability, and temperature range of the formed mesophases were affected by the terminal group length. Finally, the geometrical parameters of the prepared derivatives enhanced the production of a broad N mesomorphic range.

The mesomorphic properties of rod-like molecules are influenced by their aspect ratio, dipole moment, polarizability, and competitive contact between terminal moieties. That is, mesomeric configurations influence molecular geometry, which has an essential effect on molecular–molecular interactions. The lateral attraction between the long alkoxy-chains (n) on the planar molecules, enforced by the molecular aggregation between calamitic molecules, has been shown to affect the thermal stability of mesophases. In addition, the end-to-end interactions between terminal flexible chains differ based on the mesomeric effects. The mesomorphic properties of components are influenced by these parameters in different ratios.

For the series **I*n***, the transition normalized entropy changes (**Δ*S***_N-I_/R) were calculated and are included in Table 1. The entropy values were observed to be in irregular correlation with the alkoxy terminal chain length n. The broad temperature ranges in the mesophase can be explained by the increase in the end-to-end aggregations of the molecules as the alkoxy chain’s terminal length increases, resulting in an irregular interaction. Furthermore, the slight entropy variations seen for the investigated compounds could be attributed to congruent weak conjugative interactions between both of the mesogenic cores that were connected in contraposition to each other to the molecule’s central azomethine group [46].

### 2.2. DFT Studies

**Figure 4:**
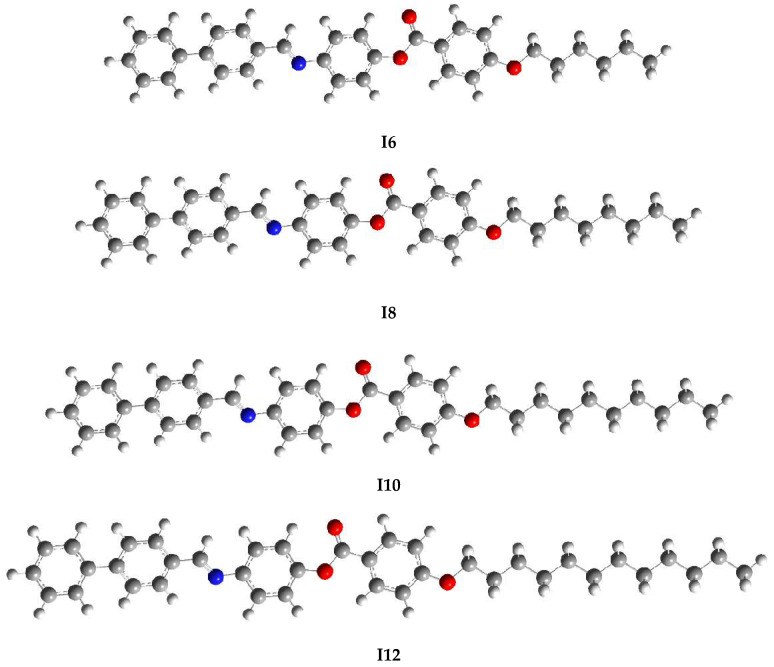
The optimized geometries of the studied compounds **I*n***.

The optimized structures of the homologous series **I6**, **I8**, **I10**, and **I12** are shown in Figure 4, and the energetics, as well as the thermodynamic parameters, are presented in Table 2. Figure 4 reveals that all structures were semilinear but with two terminal benzene moieties that were out of the molecular plane by around 32° and 36° affecting the planarity of the molecules. Planarity plays an essential role in the packing degree of the molecules in the condensed liquid crystalline phase. In addition, the coplanarity of the liquid crystals has a significant effect on the mesophase behavior. Therefore, the small twist angle of the terminal benzene rings enhanced the molecular planarity that boosted the molecular packing in the condensed liquid crystalline phase. The results showed that the geometry, specifically, the molecular planarity did not vary significantly upon adding a longer alkoxy chain. Our results show a good way of predicting the preferred molecular structure in the gas phase; however, the presence of these compounds in the condensed liquid crystalline phases may exhibit some deviations [47].

The molecular polarizabilities and dipole moments are important parameters in characterizing liquid crystalline substances by understanding and quantifying intermolecular interactions [48]. The polarizability and dipole moments for the **I*n*** series were computed at the same level of theory and are presented in Table 3. The results revealed that the polarizability increased with increasing the chain length ranging from 472 to 547 Bohr^3^. Elongated molecules have electrons that are moved easily, which increases their polarizability and, thus, strengthen the dispersion forces leading to an increase in melting and boiling points. On the other hand, small, compact, and symmetrical molecules are less polarizable with weaker dispersion forces.

**Figure 5:**
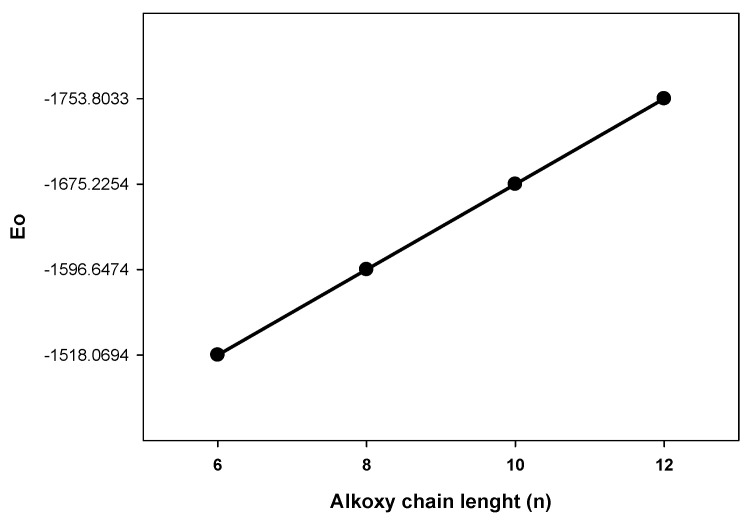
Dependence of the terminal chain length on the thermal energies of series **I*n***.

To study the effect of the terminal length on the stability of the molecules, the thermal energy of the calculated structures was correlated to the experimentally established mesophase stability values as well as the length of the alkoxy chains (Figure 5). As the length of the alkoxy chain became longer, the stability of mesophase decreased. The strong stacking of the aromatic rings as well as the aggregation of the alkoxy chains could explain these findings. In regard to the stacking of the benzene rings, the strength of the aggregation increased as the number of alkoxy chains increased.

**Figure 6:**
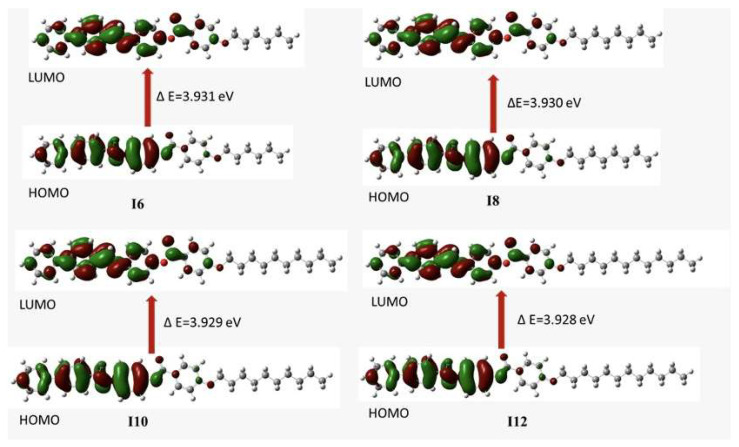
The FMOs for the prepared **I*n*** series calculated at the B3LYP/6-31+G(d,p) level of theory.

Frontier molecular orbitals (FMO) provide a reasonable qualitative indication of the excitation and electron transport properties of molecular characteristics, such as stability and optical properties. [48,49,50,51]. Molecular energy levels of the highest occupied (HOMO) and the lowest unoccupied (LUMO) orbitals determine the energy gap that reveals the characters of the molecule’s electron donors and acceptors. The HOMO–LUMO gap (ΔE) is a remarkable parameter to predict the nonlinear optical properties (NLO) of molecular systems. A low ΔE value is characteristic of materials that are less stable but easily polarizable indicating easier electronic transition and, hence, better NLO properties of a molecule [51]. A large energy gap is related to insulation properties and high molecular stability. In the case of the studied molecules, the HOMO and LUMO energy values did not show any meaningful dependence on the terminal length of the attached alkoxy chain, as their values were similar in all the studied molecules. Consequently, the energy gap width was not influenced by the polarization of terminal groups. Additionally, the absolute electronegativity, χ, chemical potentials, μ, absolute hardness, η, absolute softness, σ, global electrophilicity, ω, global softness, S, and the additional electronic charge, ΔN_max_, were calculated from the HOMO and LUMO energies (Table 3) to give indications of the stability and reactivity of the molecules studied. Hardness is one of the most common and useful parameters that help in understanding the behavior and reactivates of molecules and can be considered a measure of the stability of molecules. It is coupled with the softness, electronic chemical potential, or absolute electronegativity in this regard [52,53]. The softness (**S**) shows the photoelectric sensitivity and the degree of the polarizability of materials [54]. In addition, ΔN_max_ measures the number of electrons that can be transferred from the molecule in reaction; soft molecules would have a large value of ΔN_max_ [52]. The calculations demonstrated that the title molecules had a small HOMO–LUMO energy gap (3.9 eV); so, it is predicted that the studied molecules are soft and reactive. On the other hand, as shown in Figure 6, it is clear that the electron densities of the HOMOs and the LUMOs were mainly localized on the aromatic rings, while there was no apparent impact of the terminal chains on the distribution of the FMOs’ electron densities. It can be noticed that both frontier orbitals had the same shape in all the studied molecules.

**Figure 7:**
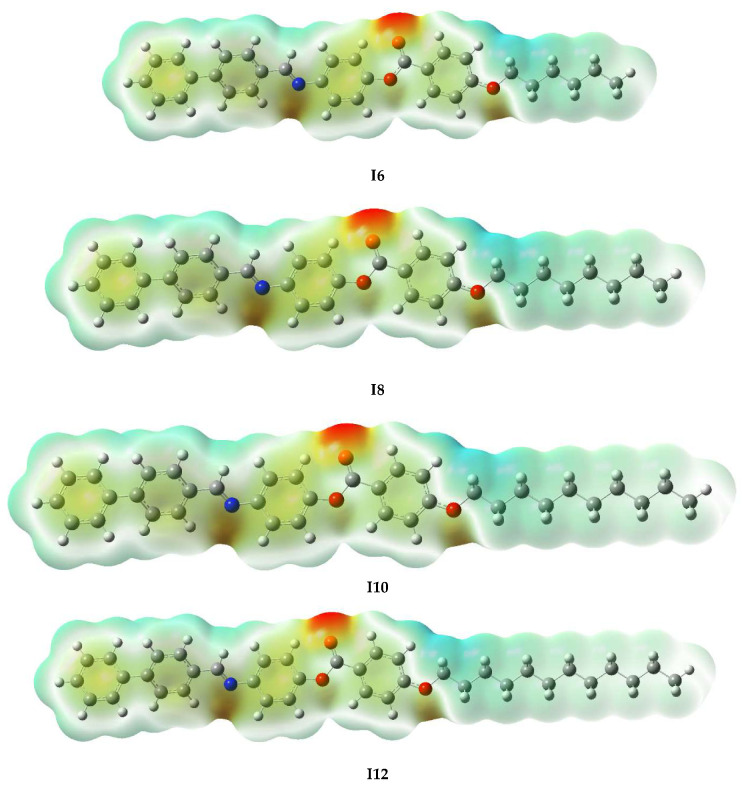
The molecular electrostatic potentials of the prepared **I6**, **I8**, **I10**, and **I12**.

The molecular electrostatic potential energy surface (MEP), which illustrates the electronic density distribution, is a valuable parameter that sheds some light on the electrophilic and nucleophilic centers in a chemical compound. Moreover, it describes the hydrogen-bonding between molecules [49,50]. The MEP of the **I*n*** series has been calculated using the geometries of the compounds optimized at B3LYP/6-31+G(d,p). The negative sites (red) of the MEP are characterized by a greater electron density and are related to the nucleophilic reactivity. The positive sites (green or blue) are the area of low electron density and are associated with the electrophilicity. The potential energy range of the potential map was found to be − 5.221e^−2^ esu to + 5.221e^−2^ esu. In all the **I*n*** series, as shown in Figure 7, the O and N centers had high electron density and represented the nucleophilic part, while low electron density was observed around the alkyl chain.

## 3. Experiments

### Synthesis

**Scheme 1:**
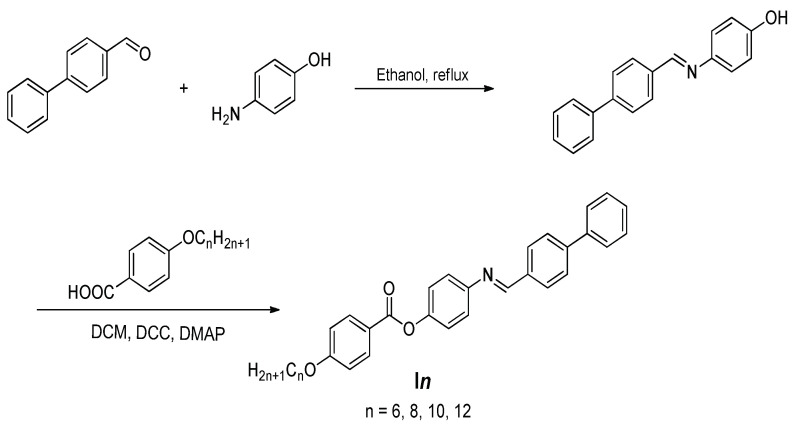
Synthesis of 4-(([1,1′-biphenyl]-4-ylmethylene)amino)phenyl 4-(alkoxy)benzoate, I*n*.

The new derivatives **I*n*** were prepared according to Scheme 1:

The details of preparations are provided in Supplementary Materials.

## 4. Computational Characterization

Computational DFT calculations [55,56] were performed at the B3LYP/6-31+G(d,p) level combination [57,58,59] using the Gaussian 16 package [60] to explore the electronic properties and energetics of the prepared homologous series (**I*n***) of the investigated liquid crystals. Geometry optimizations were employed for all the studied compounds, where all stationary points on the potential energy surface of the systems were characterized by having zero norms. The nature of the optimized geometries was investigated by frequency calculations at the same level of theory (B3LYP/6-31+G(d,p)) to verify whether those geometries were minima or transition states on the potential energy surfaces of the studied systems. We found no negative eigenvalues in the second derivatives matrices indicating that all of the obtained stationary points were minima. The thermodynamic parameters such as the electronic energy (E), enthalpy (H), entropy (S), and Gibbs free energy (G) were computed employing the B3LYP/6-31+G(d,p) level of theory. The distribution of the highest occupied and lowest unoccupied frontier molecular orbitals (HOMO and LUMO, respectively) as well as the molecular electrostatic potential (MEP) of the optimized structures were analyzed.

The reactivity parameters such as the energy gap (∆E), absolute electronegativity (χ), absolute hardness (η), chemical potentials ( μ), global electrophilicity (ω), global softness (S), absolute softness (σ), and additional electronic charge (ΔN_max_) were calculated as follows [49,50,61,62,63]:

      E_*LUMO*_—E_*HOMO*_

         μ = ½ (E_*LUMO*_ + E_*HOMO*_)

x = −μ

          η = ½ (E_*LUMO*_—E_*HOMO*_)

 σ = 1/η

  ω = Pi^2^/2 η

 S = 1/2η

      ΔN_max_ = −μ/η

## 5. Conclusions

The new biphenyl azomethine liquid crystal homologues series, (E)-4-(([1,1′-biphenyl]-4-ylmethylene)amino)phenyl 4-(alkoxy)benzoate, was examined experimentally and theoretically. FTIR, NMR, and elemental analysis investigations were utilized to affirm the chemical structures of the synthesized compounds. The mesomorphic characteristics of the present investigated homologues were carried out by DSC and POM. All of the prepared derivatives were confirmed to have high thermal mesomorphic stabilities and wide nematogenic temperature ranges with enantiotropic properties. The theoretical calculations revealed that the polarizability of the title compounds increased with the increase in the length of the terminal group, while a negligible effect of the chain length on the HOMO–LUMO energy gap was observed. The reactivity parameters such as the softness, hardness, and electrophilicity were calculated and predicted that the studied molecules are soft and reactive. All of the estimated architectures’ thermal parameters, dipole moments, and polarizability were examined. As the length of the alkoxy terminal chain varied, the relationships between the values of these parameters and mesophase stability were highlighted.

## Figures and Tables

**Table 1:** Transition temperatures of phases (°C), (enthalpy **ΔH**, kJ/mole), mesomorphic range (**ΔT**), and normalized entropy, **ΔS/R** of transition for series **I*n***.

Comp.	T_Cr-N_	ΔH_Cr-N_	T_N-I_	ΔH_N_-_I_	ΔT_N_	ΔS_N-I_/R
**I6**	135.8	43.72	244.3	1.99	108.50	0.46
**I8**	130.5	45.08	226.8	1.87	96.30	0.45
**I10**	113.5	39.88	196.2	1.95	82.70	0.50
**I12**	115.8	41.79	193.3	1.91	77.50	0.49

Cr-N = solid to nematic transition. N-I = Nematic to the isotropic phase transition.

**Table 2:** The energetics and thermodynamical parameters (in Hartree) of the optimized structures at the B3LYP/6-31+G(d,p) level of theory.

	E_0_	E_298_	H_298_	G_298_
**I6**	−1518.069423	−1518.036426	−1518.035482	−1518.141578
**I8**	−1596.647410	−1596.611662	−1596.610718	−1596.723890
**I10**	−1675.225375	−1675.186884	−1675.185940	−1675.306220
**I12**	−1753.803278	−1753.762061	−1753.761117	−1753.888488

**Table 3:** Various quantum chemical parameters for the optimized structures of the studied molecules at B3LYP/6-31+G(d,p) level of theory.

E (a.u)	I6	I8	I10	I12
Dipole moment (D)	2.359	2.402	2.426	2.442
Polarizability (α)	472.08	497.42	522.50	547.42
E_HOMO_ (eV)	−5.950	−5.948	−5.948	−5.948
E_LUMO_ (eV)	−2.019	−2.018	−2.019	−2.019
ΔE (eV)	3.931	3.930	3.929	3.929
χ (eV)	3.984	3.983	3.983	3.983
η (eV)	1.965	1.965	1.964	1.964
σ (eV^−1^)	0.508	0.508	0.509	0.509
μ (eV)	−3.984	−3.983	−3.983	−3.983
S (eV^−1^)	0.254	0.254	0.254	0.254
ω (eV)	4.038	4.036	4.038	4.038
ΔN_max_	2.027	2.027	2.028	2.028

## Data Availability

Not applicable.

## Supplementary Materials

The following supporting information can be downloaded at: https://www.mdpi.com/article/10.3390/molecules27134150/s1, Figures S1—S5.

## Sample Availability

Samples of the compounds are available from the authors.
