# Supplementary material for: Wide Nematogenic Azomethine/Ester Liquid Crystals Based on New Biphenyl Derivatives: Mesomorphic and Computational Studies
Source: Molecules. 2022 Jun 28;27(13):4150. doi: 10.3390/molecules27134150 (PMC9268434; doi:10.3390/molecules27134150)
Supplement: Supplementary file 1 [file molecules-27-04150-s001.zip › molecules-1757662-supplementary.pdf]

molecules

MDPI

Article

Supplementary Materials

1. Materials

biphenyl-4-carboxaldehyde, 4-aminophenol, 4-Hexyloxylbenzoic acid, 4-octoyloxybenzoic acid, 4-decyloxybenzoic acid, and 4-dodecyloxybenzoic acid. were purchased from Sigma Aldrich (Germany). dichloromethane, *N,N′*- dicyclohexylcarbodiimide (DCCD), ethanol and 4-dimethyl aminopyridine (DMAP) were purchased from Aldrich (Wisconsin, USA).

2. Synthesis of (E)-4-(([1,1'-biphenyl]-4-ylmethylene)amino)phenol

An equimolar amount of biphenyl-4-carboxaldehyde (5 mmol) and 4-aminophenol (5 mmol) in ethanol (20 ml) was refluxed for 70 minutes. The reaction mixture was allowed to cool, and the precipitated product was filtered. The obtained solid was recrystallized from ethanol. The melting points were determined for the prepared imines were accordance with the literature.

3. Synthesis of 4-(([1,1'-biphenyl]-4-ylmethylene)amino)phenyl 4-(alkoxy)benzoate, In

Molar equivalents of 4-(([1,1'-biphenyl]-4-ylmethylene)amino)phenol and 4-alkoxybenzoic acid (0.01 mol) were dissolved in dry methylene chloride (DCM) (25 ml). 0.02 molar of *N, N′*-dicyclohexylcarbodiimide (DCC) and a trace amount of 4-dimethyl aminopyridine (DMAP) were added to the reaction mixture. The reaction was left under stirring at room temperature for 72 hrs. The separated byproduct, dicyclohexylurea (DCU), was filtered off. The filtrate was then evaporated, and the obtained product was recrystallized from ethanol.

4. Analyses of materials

(E)-4-(([1,1'-biphenyl]-4-ylmethylene)amino)phenol

^{1}H NMR (850 MHz, DMSO) δ 9.55 (s, 1H, OH), 8.67 (s, 1H, CH=N), 7.99 (d, *J* = 8.2 Hz, 2H, Ar-H), 7.82 (d, *J* = 8.2 Hz, 2H, Ar-H), 7.75 (d, *J* = 7.3 Hz, 2H, Ar-H), 7.51 (t, *J* = 7.7 Hz, 2H, Ar-H), 7.42 (t, *J* = 7.4 Hz, 1H, Ar-H), 7.24 (d, *J* = 8.6 Hz, 2H, Ar-H), 6.83 (d, *J* = 8.5 Hz, 2H, Ar-H). ^{13}C NMR (214 MHz, DMSO) δ 157.08, 156.83, 143.05, 142.75, 139.82, 136.04, 129.53, 129.34, 128.45, 127.44, 127.24, 123.05, 116.20.

(E)-4-(([1,1'-biphenyl]-4-ylmethylene)amino)phenyl 4-(hexyloxy)benzoate

^{1}H NMR (850 MHz, DMSO) δ 8.73 (s, 1H, CH=N), 8.09 (d, *J* = 8.7 Hz, 2H, Ar-H), 8.05 (d, *J* = 8.2 Hz, 2H, Ar-H), 7.86 (d, *J* = 8.2 Hz, 2H, Ar-H), 7.78 (d, *J* = 8.2 Hz, 2H, Ar-H), 7.52 (t, *J* = 7.7 Hz, 2H, Ar-H), 7.43 (t, *J* = 7.3 Hz, 1H, Ar-H), 7.40 (d, *J* = 8.6 Hz, 2H, Ar-H), 7.32 (d, *J* = 8.6 Hz, 2H, Ar-H), 7.13 (d, *J* = 8.8 Hz, 2H, Ar-H), 4.10 (t, *J* = 6.5 Hz, 2H, OCH_2), 1.76 (dt, *J* = 14.3, 8.3 Hz, 2H, CH_2), 1.44 (dt, *J* = 15.0, 7.5 Hz, 2H, CH_2), 1.36 – 1.26 (m, 4H, $2CH_2$), 0.89 (t, *J* = 7.0 Hz, 3H, CH_3). ^{13}C NMR (214 MHz, DMSO) δ 132.51, 129.85, 129.58, 127.53, 127.31, 123.15, 122.55, 115.18, 68.43 (OCH_2), 31.38 (CH_2), 28.92 (CH_2), 25.45 (CH_2), 22.52 (CH_2), 14.32 (CH_3).

(E)-4-(([1,1'-biphenyl]-4-ylmethylene)amino)phenyl 4-(dodecyloxy)benzoate

^{1}H NMR (850 MHz, DMSO) δ 8.73 (s, 1H, CH=N), 8.09 (d, *J* = 8.7 Hz, 2H, Ar-H), 8.05 (d, *J* = 8.2 Hz, 2H, Ar-H), 7.86 (d, *J* = 8.1 Hz, 2H, Ar-H), 7.78 (d, *J* = 8.1 Hz, 2H, Ar-H), 7.55 – 7.50 (m, 2H, Ar-H), 7.45 – 7.42 (m, 1H, Ar-H), 7.40 (d, *J* = 8.5 Hz, 2H, Ar-H), 7.32 (d, *J* = 8.5 Hz, 2H, Ar-H), 7.13 (d, *J* = 8.8 Hz, 2H, Ar-H), 4.14 – 4.06 (m, 2H, OCH_2), 1.84-1.82 (m, 2H, CH_2), 1.76 – 1.73(m, 2H, CH_2), 1.66-1.62 (m, 2H, CH_2), 1.51 (m, 2H, CH_2), 1.43-1.40 (m, 2H, CH_2), 1.32 – 1.19 (m, 2H, CH_2), 0.91 – 0.83 (m, 3H, CH_3).

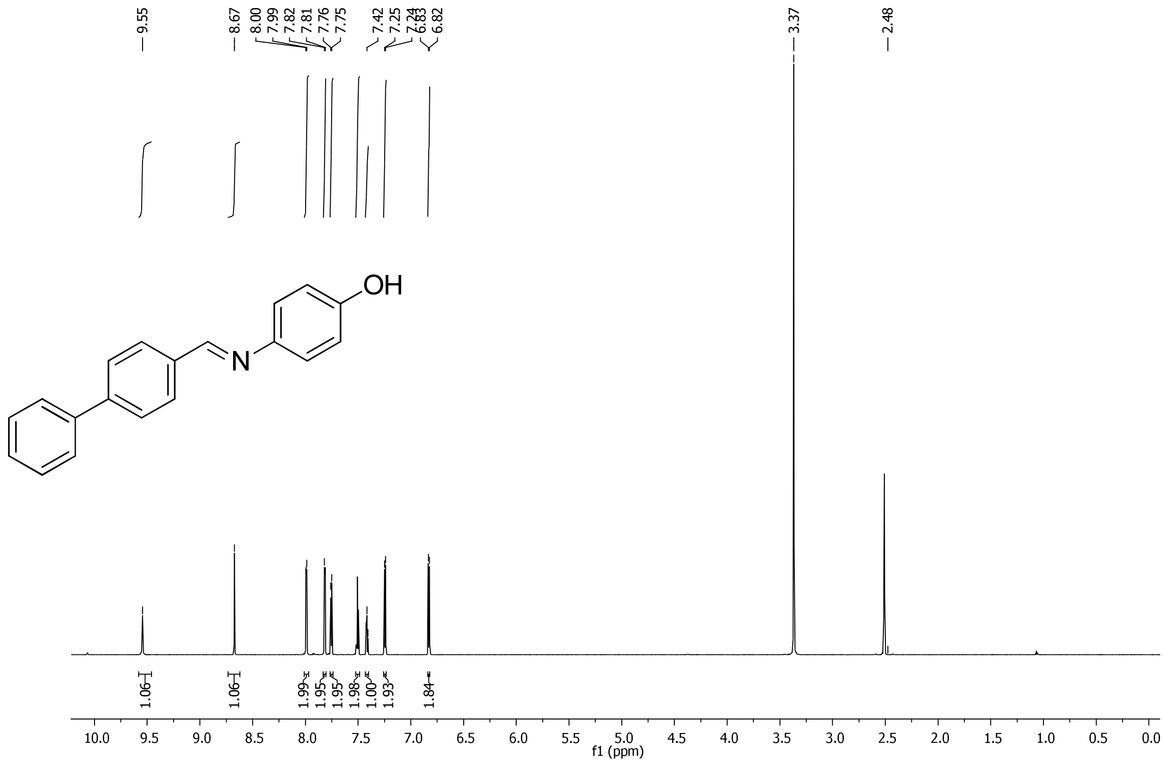

Figure S1. ^{1}H-NMR of (E)-4-(([1,1'-biphenyl]-4-ylmethylene)amino)phenol.

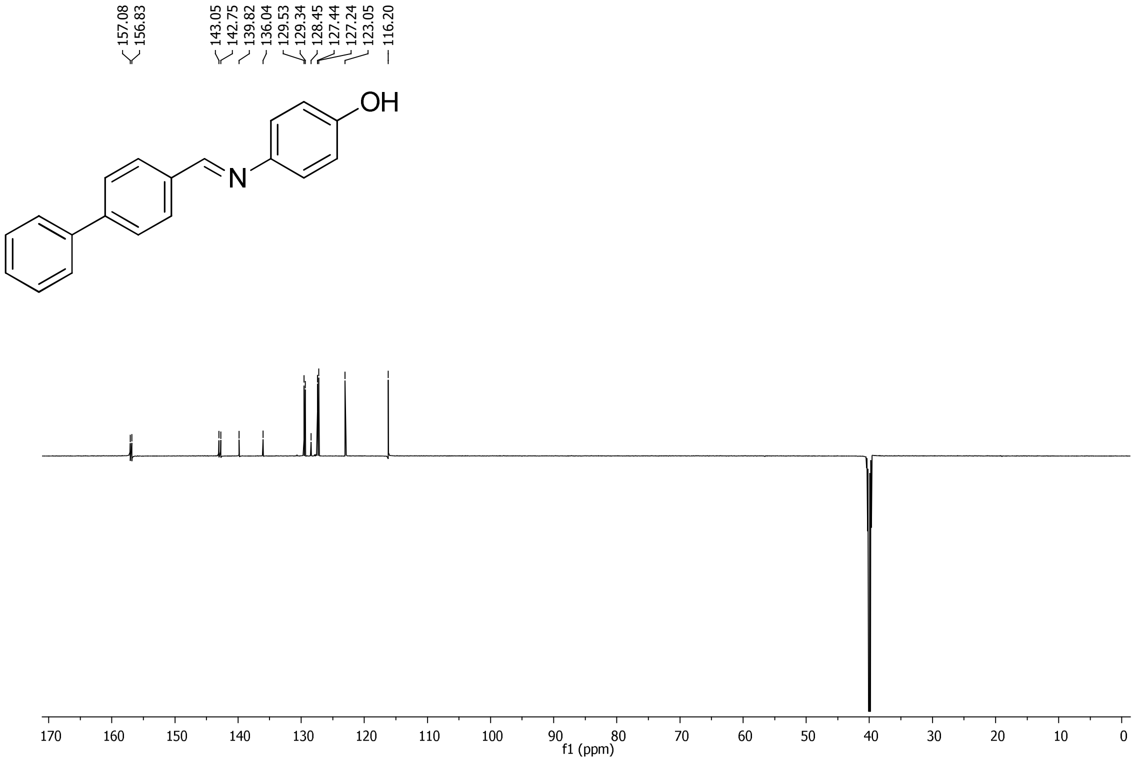

Figure S2. ^{13}C-NMR of (E)-4-(([1,1'-biphenyl]-4-ylmethylene)amino)phenol.

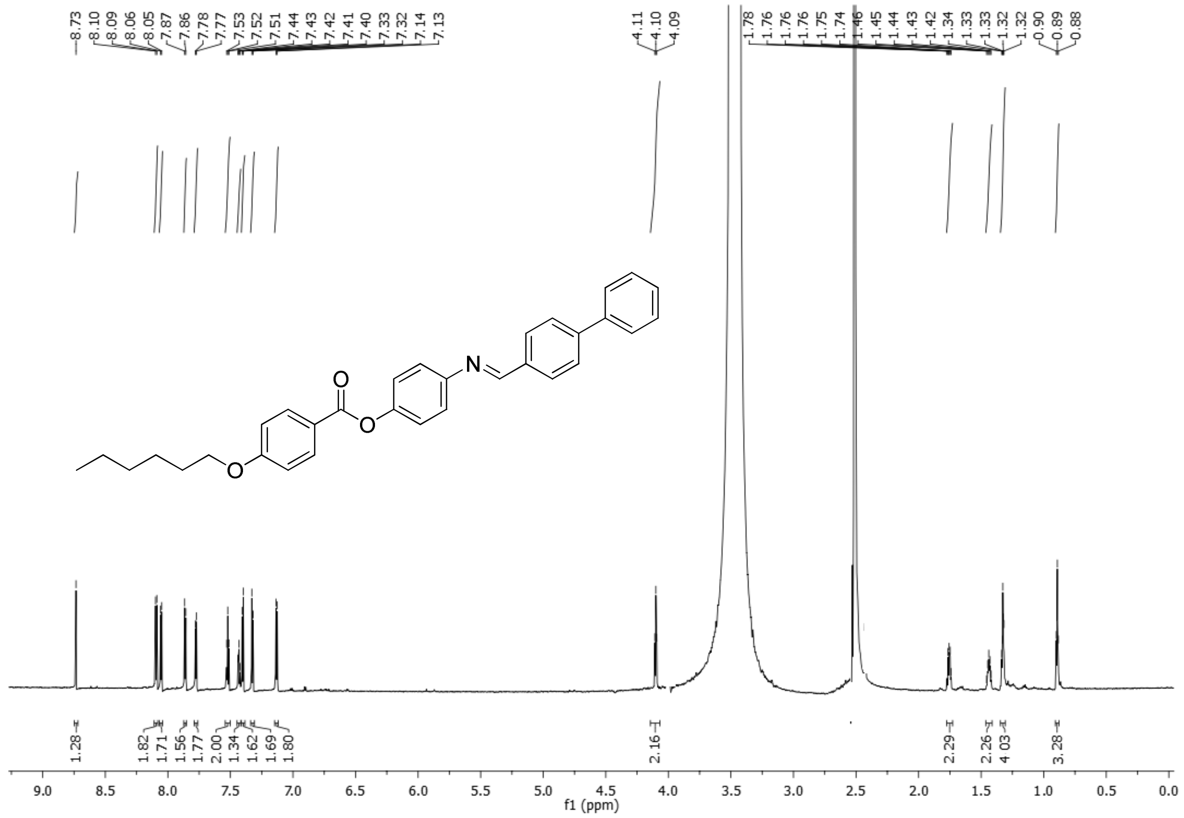

Figure S3. ^{1}H-NMR of E)-4-(([1,1'-biphenyl]-4-ylmethylene)amino)phenyl 4-(hexyloxy)benzoate).

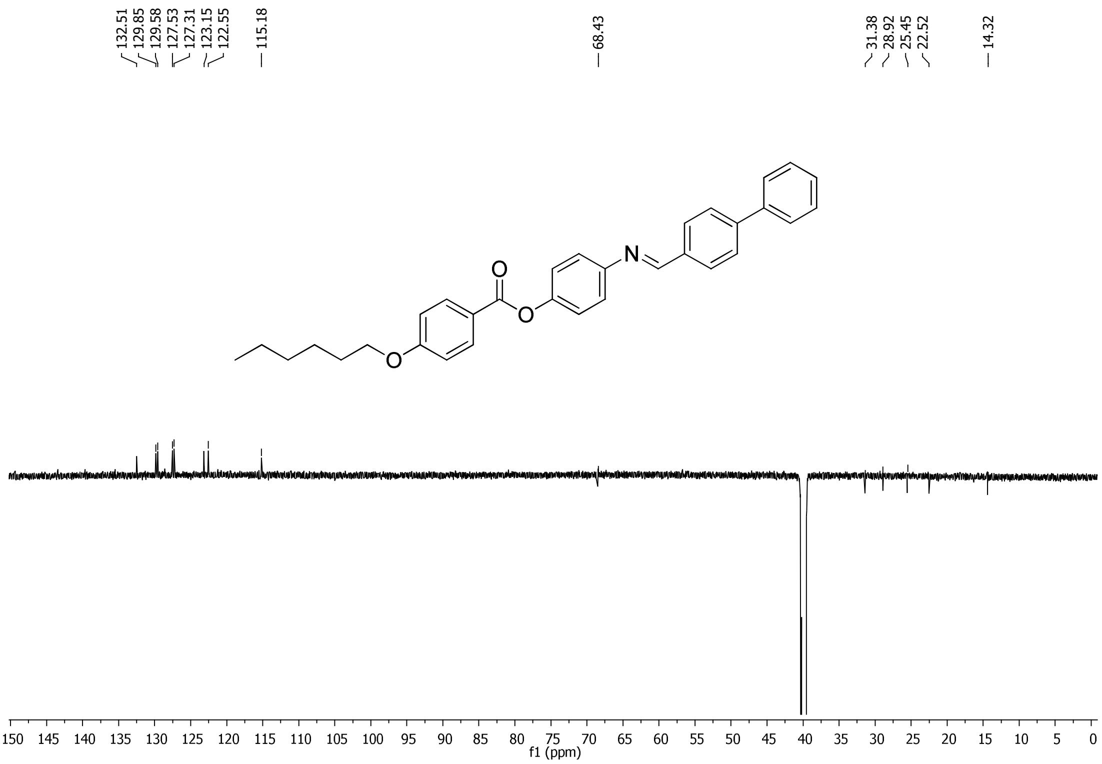

Figure S4. ^{13}C-NMR of E)-4-(([1,1'-biphenyl]-4-ylmethylene)amino)phenyl 4-(hexyloxy)benzoate).

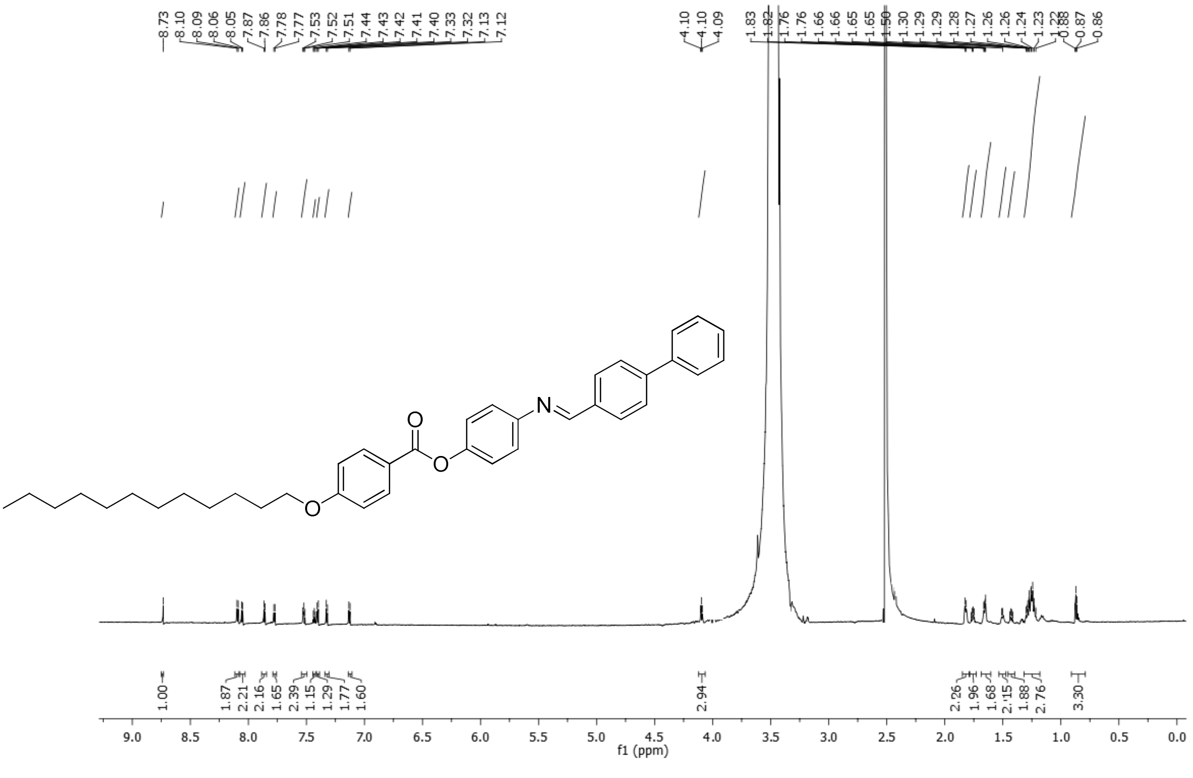

Figure S5. ^{1}H-NMR of (E)-4-(([1,1'-biphenyl]-4-ylmethylene)amino)phenyl 4-(dodecyloxy)benzoate.

5.Charactrizations:

Melting points were determined by MEL-TEMP II melting point apparatus in open glass capillaries and were uncorrected. The IR spectra were recorded as potassium bromide (KBr) discs on a Perkin-Elemer FT-IR (Fourier-Transform Infrared Spectroscopy), college of Science, Taibah University. The NMR spectra were carried out at ambient temperature (~25 °C) on a (JEOL) 500 MHz spectrophotometer using tetra methyl silane (TMS) as an internal standard, NMR Unit, Faculty of Science, Mansoura University. Chemical shift was recorded as δ values in parts per million (ppm), and the signals were reported as s (singlet), d (doublet), t (triplet) and m (multiplet). Elemental analyses were analyzed at the Micro analytical Unit, Faculty of Science, Cairo University.

TA Instruments Co. (Q20 Differential Scanning Calorimeter, DSC; USA) was used for recording phase transitions. DSC calibration was carried using lead and indium melting temperature and enthalpy. Samples of 2–3 mg were used in aluminum pans for DSC investigation. The heating rate was 10°C/min in nitrogen gas as an inert atmosphere (30 ml/min). All transitions measured were for the second heating scan.

Transition temperatures for the prepared compounds were checked and phases identified by Polarized optical microscope (POM, Wild, Germany) attached with Mettler FP82HT hot stage.
